# Work Hours, Stress, and Burnout Among Resident Physicians

**DOI:** 10.1001/jamanetworkopen.2025.53974

**Published:** 2026-01-14

**Authors:** Sydney F. Tan, Hana Siddiqui, Alex Pinto, Bridget Paur, Guanhua Chen, Dawn M. Elfenbein, Vincent Minichiello, Bruce Barrett, Richard J. Davidson, Simon B. Goldberg

**Affiliations:** 1 Department of Surgery, University of Wisconsin School of Medicine and Public Health, Madison; 2 University of Wisconsin School of Medicine and Public Health, Madison; 3 Department of Biostatistics and Medical Informatics, University of Wisconsin School of Medicine and Public Health, Madison; 4 Department of Family Medicine and Community Health, University of Wisconsin School of Medicine and Public Health, Madison; 5 Center for Healthy Minds, University of Wisconsin-Madison, Madison; 6 Department of Psychology, University of Wisconsin-Madison, Madison; 7 Department of Psychiatry, University of Wisconsin School of Medicine and Public Health, Madison; 8 Department of Counseling Psychology, University of Wisconsin-Madison, Madison

## Abstract

**Importance:**

Burnout is highly prevalent among resident physicians, producing serious personal, professional, and system consequences. While work hour restrictions were implemented to improve patient safety and resident fatigue, the relationship between work hours with burnout and well-being remains unclear.

**Objective:**

To evaluate the association of work hours with burnout, stress, and self-perceived competency among residents in high-burnout specialties, and to explore potential demographic and well-being–related moderators.

**Design, Setting, and Participants:**

This cross-sectional study included responses from a remote survey conducted from February to June 2024 as part of a randomized clinical trial evaluating a well-being intervention among resident physicians. Eligible participants were residents enrolled in high-burnout specialty residency programs (surgery, obstetrics-gynecology, family, internal, and emergency medicine) in the US.

**Exposure:**

Self-reported average weekly work hours and total hours worked in the past week.

**Main Outcome and Measures:**

Burnout, stress, and self-assessed Accreditation Council for Graduate Medical Education (ACGME) competency milestones, adjusting for demographics.

**Results:**

A total of 540 residents responded (356 cisgender women [66.8%]; 112 Asian [21.1%], 28 Black [5.3%], 355 White [67.0%]). The sample was predominantly in a medical specialty (56%). The mean (SD) number of average hours worked was 65.4 (11.3) and the mean (SD) number of hours worked in the last week was 60.1 (17.4). There was no significant association between burnout and average hours worked (β = 0.05 [95% CI, −0.05 to 0.15]; *P* = .34) or hours worked last week (β = 0.06 [95% CI, −0.03 to 0.15]; *P* = .21). However, stress was positively associated with average hours worked (β = 0.17 [95% CI, 0.07 to 0.26]; *P* = .001) and hours worked last week (β = 0.27 [95% CI, 0.18 to 0.36]; *P* < .001). Self-assessed ACGME competency milestones were also positively associated with average hours worked (β = 0.14 [95% CI, 0.07 to 0.21]; *P* < .001) and hours worked last week (β = 0.08 [95% CI, 0.01 to 0.15]; *P* = .02). Despite exploring a large set of candidate moderators, very few moderators were identified and none survived *P* value adjustment.

**Conclusion and Relevance:**

In this cross-sectional nationwide study of resident physicians in high-burnout specialties, longer work hours were associated with higher stress and self-perceived competency, but not with burnout. This suggests that work hours alone may not explain high burnout levels in residency; a more comprehensive approach beyond work hour restrictions is needed to support resident well-being in training.

## Key Points

**Question:**

Are work hours associated with stress, burnout, and self-perceived competency among residents in high-burnout specialties?

**Findings:**

In this cross-sectional study including a survey sample of 540 US residents in high-burnout specialties, longer work hours were significantly associated with higher stress and higher self-perceived competency, but work hours were not significantly associated with burnout.

**Meaning:**

These findings suggest that further restricting work hours may not reduce burnout and could negatively affect residents’ perceived competency, highlighting the need for more comprehensive strategies to support well-being beyond work hours alone.

## Teaser

This cross-sectional study of US medical residents in high-burnout specialties examines the association of longer work hours with stress, burnout, and self-perceived competency.

## Introduction

Burnout is highly prevalent among resident physicians, especially those on the front lines of care.^1,2,3^ While stress is often a temporary reaction to job demands, burnout represents a pathologic state of prolonged, intense stress that leads to exhaustion, cynicism, and reduced sense of accomplishment with widespread consequences.^2^ Resident burnout is linked to higher rates of divorce, depression, and suicide, as well as increased incidence of medical errors, attrition, and absenteeism.^4,5,6^

Residency involves heavy clinical demands, research, teaching, and administrative duties that exacerbate stress and burnout. In 2003, concerns about fatigue and patient safety ultimately led to the Accreditation Council for Graduate Medical Education (ACGME) to require programs to restrict resident duty hours to a maximum monthly average of 80 hours per week, with no shifts exceeding 24 consecutive hours.^7^ Subsequently, work hour restrictions have raised concerns about the quality of clinical education and clinical competency of residents.^8,9^ More recently, the conversation around work hour restrictions has included concerns about resident physician well-being and burnout.^9,10^ However, studies examining the effects of work hour restrictions on resident burnout have been conflicting.^11,12,13^

Utilizing a nationwide sample of resident physicians in high-burnout specialties, this study aims to evaluate the association between work hours and stress, burnout, and self-perceived competency. Additionally, the study explores potential moderators of these associations, including well-being and demographic factors.

## Methods

### Study Design

In 2024, a nationwide, cross-sectional survey was conducted from February 21 to June 26 as part of the baseline assessment in a randomized clinical trial evaluating a meditation-based well-being training for residents (ClinicalTrials.gov NCT06149156). The study was approved by the minimal risk research institutional review board at the University of Wisconsin-Madison. Participants provided electronic informed consent. Recruitment methods included residency program director listservs, social media platforms, and resident word-of-mouth. This study followed the Strengthening the Reporting of Observational Studies in Epidemiology (STROBE) reporting guideline.

### Participants

Eligible participants included residents enrolled in a US residency program in high-burnout specialties^1,3^ (general surgery and surgical subspecialties, obstetrics-gynecology, family, internal, and emergency medicine). Exclusion criteria included residents in a nontarget specialty, regular meditation practice within the prior six months, and no previous use of the publicly available, well-being training being studied (The Healthy Minds Program app).^14^ Participants were paid $100 for completing the randomized trial, but no compensation was offered for completing the baseline survey specifically.

### Outcomes

We collected demographic data on gender, race, ethnicity, specialty, training level, relationship status, geography, time of survey completion, average hours worked per week, and hours worked in the last week. The survey was administered electronically via REDCap, a secure, web-based platform.^15^

Burnout, well-being, and competency outcomes were collected through validated self-report instruments. Burnout was measured using the 9-item abbreviated Maslach Burnout Inventory-Human Services Survey depersonalization and emotional exhaustion subscales (MBI-HSS; range, 0-36; higher scores indicate higher burnout).^16,17^ Personal accomplishment was measured using the MBI-HSS personal accomplishment subscale (range, 0-18; higher scores indicate higher personal accomplishment and lower burnout).^16,17^ Stress was measured using the 10-item Perceived Stress Scale (PSS; range, 0-40; higher scores indicate higher perceived stress).^18^ Self-perceived competency was assessed using self-assessment on ACGME milestones in core competencies of patient care, practice-based learning and improvement, professionalism, and interpersonal and communication skills (range, 1-5; higher scores indicate higher self-perceived competency).^19^

Other well-being-related outcomes included National Institutes of Health (NIH) Toolbox Loneliness (range, 1-5; higher scores indicate greater loneliness),^20^ PROMIS Sleep Disturbance (scored as T-scores, mean [SD] 50 [10]; higher scores indicate more sleep disturbance),^21^ Flourishing Index (range, 0-10; higher scores indicating higher flourishing),^22^ Brief Resilience Scale (range, 0-6; higher scores indicating higher resilience),^23^ PROMIS Meaning and Purpose (scored as T-scores, mean [SD], 50 [10]; higher scores indicate more meaning and purpose in life),^24^ Five Facet Mindfulness Questionnaire (FFMQ) Acting with Awareness Subscale (range, 1-5; higher scores indicate greater ability to act with awareness),^25^ and FFMQ Nonjudging Subscale (range, 1-5; higher scores indicate greater nonjudging, the ability to take a non-evaluative stance toward inner experiences).^25^

### Statistical Analysis

For use in analyses, specialties were grouped into medicine (emergency, family, and internal medicine) and surgery (general, subspecialties, and obstetrics-gynecology). Training level was coded by specialty into intern (postgraduate year [PGY] 1), midlevel (medicine PGY 2, surgery PGY 2-3) and senior (medicine PGY 3 or more, surgery PGY 4 or more). Race categories with small numbers (multiple races, American Indian or Alaskan Native, and Native Hawaiian or Pacific Islander) were coded as multiple races or other. Ethnicity was analyzed as Hispanic or Latino vs non-Hispanic or Latino. Race and ethnicity were self-reported (eAppendix).

Multiple linear regression analyses were performed to assess the relationship between work hours and burnout, stress, personal accomplishment, and self-assessed ACGME competency milestones while controlling for demographic variables (gender, race, ethnicity, specialty, training level, relationship status, geography, and season of survey completion). Average hours worked and hours worked last week were run as separate models. Standardized coefficients were estimated to support interpretation and comparison across models. Multiple linear regression models and multivariate regression models were used to investigate potential moderators of the relationship between work hours and outcomes (ie, testing interactions between work hours and well-being and demographic characteristics). All statistical tests were 2-sided with statistical significance defined as *P* < .05 unless otherwise noted for multiple-comparison adjustments. Multicollinearity was evaluated using Variance Inflation Factor (VIF) scores (VIF scores for variables in eTable 9 in Supplement 1).^26^ To address the increased risk of Type 1 errors, the Benjamini-Hochberg (BH) adjustment was used to control the False Discovery Rate (FDR).^27^ All statistical analyses and figures were generated using SAS software version 9.4 (SAS Institute Inc). As less than 5% of data were missing for all study variables, missingness was handled using pairwise deletion.^28^ Sensitivity analyses was conducted by excluding participants whose work hours were 3 standard deviations from the mean (average work hours and hours worked last week) for the multiple linear regression analyses assessing the association between work hours with burnout, stress, personal accomplishment, and self-assessed ACGME competency milestones.

## Results

### Enrollment and Participant Characteristics

From February to June 2024, 926 residents were screened. Of those eligible, 195 were excluded for partially overlapping reasons: 132 were not in a target specialty, 163 had a regular meditation practice, and 156 had previously used the intervention app. After screening, 540 completed the baseline assessment. Among participants, 356 (66.8%) identified as cisgender women, 168 (31.5%) as cisgender men, and 9 (1.7%) transgender or nonbinary. This sample had a higher proportion of women compared with ACGME Resident Data 2022-2023 (consisting of the same specialties), in which 47.2% identified as female, 52.5% as male, and 0.3% as nonbinary or not reported.^29^

In terms of race and ethnicity, 355 participants (67.0%) identified as White, 112 (21.1%) as Asian, 28 (5.3%) Black, 35 (6.6%) multiple races or other, and 37 (7.0%) Hispanic. Compared with ACGME data of the same specialties, this sample included more White and non-Hispanic residents (ACGME data: White, 67.0% vs 52.8%; Hispanic, 7.0% vs 9.8%).^29^ By specialty, 303 participants (56.1%) were in a medical specialty and 43.9% were in a surgical specialty (Table 1), whereas ACGME data reported 66.2% in medical specialties and 33.8% in surgical specialties studied.^29^ The mean (SD) number of average hours worked was 65.4 (11.3) and the mean (SD) number of hours worked in the last week was 60.1 (17.4).

**Table 1.** Demographic Characteristics

Demographic	Overall, No. (%) (N = 540)
Work hours, mean (SD), h/wk	
Average hours worked	65.4 (11.3)
Hours worked last wk	60.1 (17.4)
Medicine specialty	303 (56.1)
Emergency medicine	39 (7.2)
Family medicine	155 (28.7)
Internal medicine	109 (20.2)
Surgery specialty	237 (43.9)
Cardiothoracic	4 (0.7)
General	87 (16.1)
Neurosurgery	4 (0.7)
Obstetrics and gynecology	71 (13.1)
Ophthalmology	3 (0.6)
Orthopedic	12 (2.2)
Otolaryngology	21 (3.9)
Plastics	18 (3.3)
Urology	11 (2.0)
Vascular	6 (1.1)
Gender	
Cisgender man	168 (31.5)
Cisgender woman	356 (66.8)
Transgender or nonbinary	9 (1.7)
NR	7 (1.3)
Level of training	
Intern	179 (33.3)
Midlevel resident	225 (41.9)
Senior resident	133 (24.8)
NR	3 (0.6)
Race	
Asian	112 (21.1)
Black	28 (5.3)
White	355 (67.0)
Multiple races or other^a^	35 (6.6)
NR	10 (1.9)
Ethnicity	
Hispanic or Latino	37 (7.0)
Non-Hispanic or Latino	492 (91.0)
NR	11 (2.0)
Relationship status	
Single	157 (29.2)
Married or long-term relationship	380 (70.8)
NR	3 (0.6)
Geography	
Northeast	102 (19.4)
Midwest	182 (34.5)
South	148 (28.1)
West	95 (18.0)
NR	13 (2.4)
Season	
Spring	440 (81.5)
Summer	57 (10.6)
Winter	43 (8.0)

Abbreviations: NR, not reported.

^a^ Race categories with small numbers (multiple races, American Indian or Alaskan Native, and Native Hawaiian or Pacific Islander) were coded as multiple races or other.

### Work Hours and Burnout

**Figure 1.**
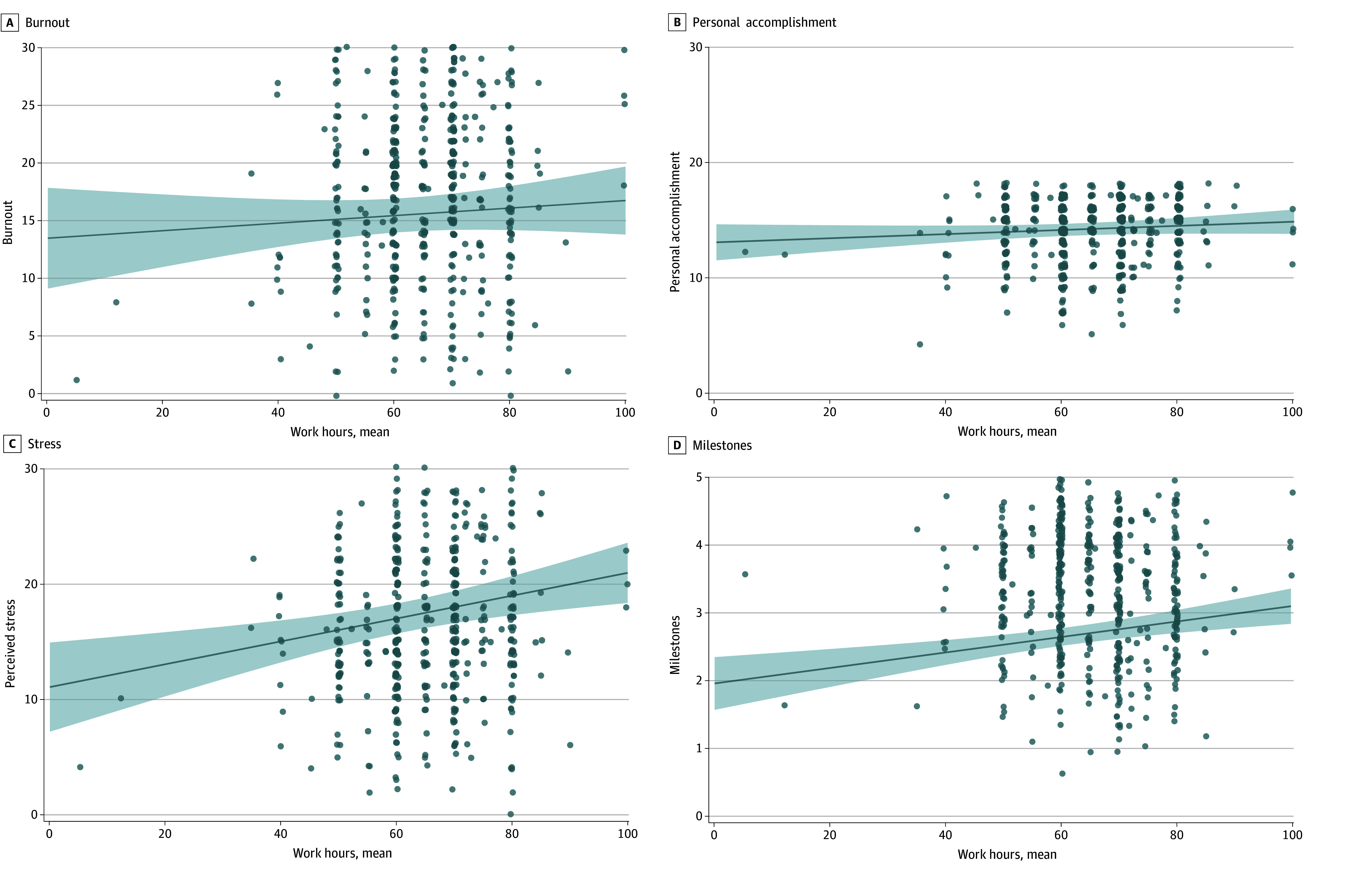
Association of Average Work Hours and Burnout, Personal Accomplishment, Stress, and Milestones^e^ ^a^Abbreviated Maslach Burnout Inventory-Human Services Survey (MBI-HSS) Emotional Exhaustion and Depersonalization subscale range: 0-36. Higher scores indicate higher burnout. ^b^MBI-HSS Personal Accomplishment subscale range: 0-18. Higher scores indicate higher personal accomplishment and lower burnout. ^c^Perceived Stress Scale (PSS) range: 0-40. Higher scores indicate greater perceived stress. ^d^The Accreditation Council for Graduate Medical Education Milestones range: 1-5. Higher scores indicate higher self-assessment of core competencies in Patient Care, Practice-Based Learning and Improvement, Professionalism, and Interpersonal and Communication Skills. ^e^Linear regression models examining the association between work hours and each outcome variable, controlling for gender, race, ethnicity, specialty, training level, relationship statues, geography, and season of survey completion.

**Figure 2.**
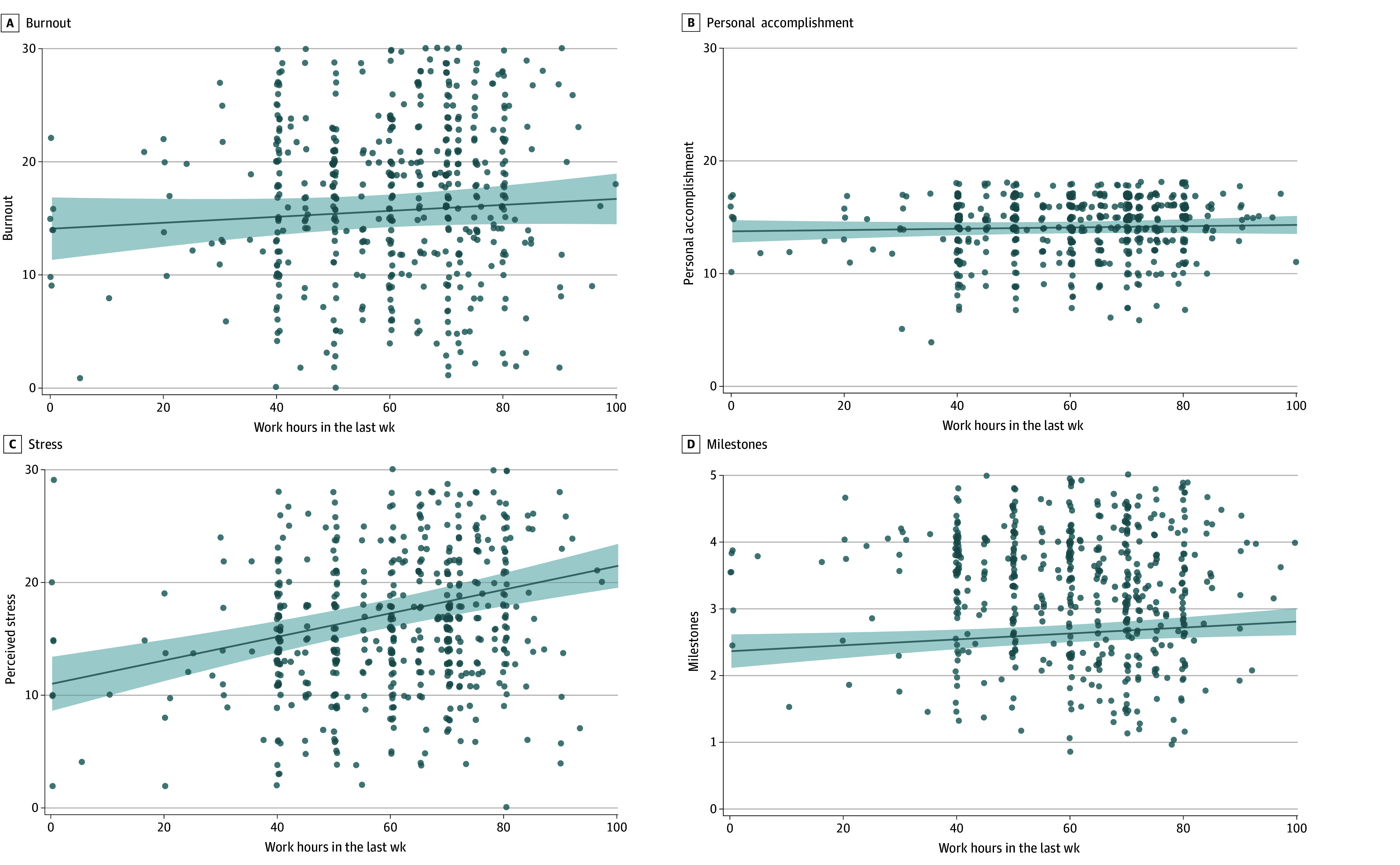
Association of Work Hours Last Week and Burnout, Personal Accomplishment, Stress, and Milestones^e^ ^a^Abbreviated Maslach Burnout Inventory-Human Services Survey (MBI-HSS) Emotional Exhaustion and Depersonalization subscale range: 0-36. Higher scores indicate higher burnout. ^b^MBI-HSS Personal Accomplishment subscale range: 0-18. Higher scores indicate higher personal accomplishment and lower burnout. ^c^Perceived Stress Scale (PSS) range: 0-40. Higher scores indicate greater perceived stress. ^d^The Accreditation Council for Graduate Medical Education Milestones range: 1-5. Higher scores indicate higher self-assessment of core competencies in Patient Care, Practice-Based Learning and Improvement, Professionalism, and Interpersonal and Communication Skills. ^e^Linear regression models examining the association between work hours and each outcome variable, controlling for gender, race, ethnicity, specialty, training level, relationship statues, geography, and season of survey completion.

There was no significant association between burnout and average hours worked (β = 0.05 [95% CI, −0.05 to 0.15]; *P* = .34) or hours worked last week (β = 0.06 [95% CI, −0.03 to 0.15]; *P* = .21) in linear regression models when controlling for demographics (Table 2; Figures 1 and 2). There were no significant moderators of this association in multiple linear regression models controlling for covariates (multilinear regression analysis of work hours and burnout moderators available in eTable 1 in Supplement 1).

**Table 2.** Association of Work Hours and Burnout, Personal Accomplishment, Stress, and Milestones^a^

Measure	Coefficient, b (95% CI)	Standardized coefficient, β (95% CI)	*P* value
**Burnout**			
Average work hours	0.33 (−0.34 to 1.00)	0.05 (−0.05 to 0.15)	.34
Work hours last wk	0.26 (−0.15 to 0.67)	0.06 (−0.03 to 0.15)	.21
**Personal accomplishment**			
Average work hours	0.18 (−0.06 to 0.42)	0.08 (−0.03 to 0.18)	.14
Work hours last wk	0.06 (−0.09 to 0.20)	0.04 (−0.06 to 0.13)	.446
**Stress**			
Average work hours	0.99 (0.40 to 1.59)	0.17 (0.07 to 0.26)	.001
Work hours last wk	1.05 (0.69 to 1.40)	0.27 (0.18 to 0.36)	<.001
**Milestones**			
Average work hours	0.11 (0.05 to 0.17)	0.14 (0.07 to 0.21)	<.001
Work hours last wk	0.04 (0.01 to 0.08)	0.08 (0.01 to 0.15)	.02

^a^ Linear regression models examining the association between work hours and each outcome variable, controlling for gender, race, ethnicity, specialty, training level, relationship status, geography, and season of survey completion. Separate models were run for average hours worked and hours worked in the last week. Coefficients (b) represent unstandardized estimates for the effect per 10-hour increase in weekly work hours (eg, for every 10-hour increase in average work hours, the Milestones score increased by 0.11). Standardized coefficients (β) allow for comparison of effect sizes across variables.

^b^ Abbreviated Maslach Burnout Inventory-Human Services Survey (MBI-HSS) Emotional Exhaustion and Depersonalization subscale range: 0-36. Higher scores indicate higher burnout.

^c^ MBI-HSS Personal Accomplishment subscale range: 0-18. Higher scores indicate higher personal accomplishment and lower burnout.

^d^ Perceived Stress Scale (PSS) range: 0-40, higher scores indicate greater perceived stress.

^e^ The Accreditation Council for Graduate Medical Education Milestones range: 1-5, higher scores indicate higher self-assessment of core competencies in Patient Care, Practice-Based Learning and Improvement, Professionalism, and Interpersonal and Communication Skills.

For personal accomplishment, which is measured on the abbreviated Maslach Burnout Inventory’s personal accomplishment subscale (range, 0-18; higher scores indicate higher personal accomplishment and lower burnout),^16,17^ there was also no significant association between personal accomplishment and average hours worked (β = 0.08 [95% CI, −0.03 to 0.18]; *P* = .14) or hours worked last week (β = 0.04 [95% CI, −0.06 to 0.13], *P* = .45) after controlling for demographics (Table 2; Figures 1 and 2). There were no significant moderators of this relationship in multiple linear regression models (multilinear regression of work hours and personal accomplishment moderators available in eTable 2 in Supplement 1).

### Work Hours and Stress

Stress was positively associated with average hours worked (β = 0.17 [95% CI, 0.07 to 0.26]; *P* = .001) and hours worked last week (β = 0.27 [95% CI, 0.18 to 0.36]; *P* < .001) after controlling for demographics (Table 2, Figures 1 and 2). For every 10-hour increase in average work hours, stress increased by 0.99 (95% CI, 0.40, 1.59; *P* < .001) and for every 10-hour increase in hours worked last week, the stress score increased by 1.05 (95% CI, 0.69 to 1.40; *P* < .001) (PSS range, 0-40; higher scores indicate higher perceived stress) (Table 2).

Resilience moderated the association between average work hours and stress in the unadjusted model, indicating that those with higher resilience were less likely to have an association between work hours and stress (β = −0.06 [95% CI, −0.11 to −0.01]; *P* = .01). However, this association did not survive FDR correction (multilinear regression of work hours and stress moderators available in eTable 3 in the Supplement 1; moderation of resilience on work hours and stress available in eFigure 1 in the Supplement 1).

### Work Hours and Milestones

Self-assessed ACGME competency milestones were positively associated with average work hours (β = 0.14 [95% CI, 0.07 to 0.21]; *P* < .001) and hours worked last week (β = 0.08 [95% CI, 0.01 to 0.15], *P* = .020) after controlling for demographics (Table 2; Figures 1 and 2). For every 10-hour increase in average work hours, the milestones score increased by 0.11 (95% CI, 0.05 to 0.17; *P* < .001) and for every 10-hour increase in hours worked last week, the milestones score increased by 0.04 (95% CI, 0.01 to 0.08; *P* = .02) (ACGME Milestones range, 1-5; higher scores indicate higher self-assessment of core competencies) (Table 2).

In the unadjusted models, sleep disturbance moderated the association of hours worked last week on milestones (β = 0.0004 [95% CI, 0 to 0.0008]; *P* = .04), with the association of work hours on milestones as stronger among those with higher sleep disturbance (multilinear regression of work hours and milestones moderators available in eTable 4 in Supplement 1, moderation of sleep disturbance on work hours and milestones available in eFigure 2 in Supplement 1). Asian race also moderated the association of hours worked last week on milestones (β = −0.01 [95% CI, −0.02 to 0]; *P* = .03), with the association of work hours on milestones weaker among Asian participants compared to White participants (for moderation of race on work hours and milestones, eTable 4 and eFigure 3 in Supplement 1). However, the moderation by sleep disturbance and race did not survive FDR correction (eTable 4 in Supplement 1).

Multivariate regression models that included all moderators and interactions matched the conclusion of the linear regression models where no interaction remained statistically significant after FDR correction (eTables 5-8 in Supplement 1). Significance tests from the sensitivity analyses removing outliers matched those in the primary analyses (eTable 10 in Supplement 1).

## Discussion

This nationwide, cross-sectional study of residents in high-burnout specialties had 3 key findings. First, there was no significant link between work hours and burnout detected in this study. Second, work hours were significantly associated with stress, indicating that longer hours may contribute to higher stress levels. Third, a positive association was observed between work hours and self-assessed ACGME competency milestones. Collectively, these findings suggest that while longer work hours may be associated with increased stress and greater self-perceived competency, work hours may not be linked to burnout.

Prior work evaluating the relationship between work hour restrictions and burnout have provided conflicting accounts on whether work hours are linked to burnout.^11,13,30^ A meta-analysis of randomized clinical trials^11^ suggested potential benefits of work hour restrictions on resident burnout; however, the overall certainty of evidence in this meta-analysis (ie, GRADE) was low or very low, with very small effect sizes, leaving associations uncertain. The present study contributes to this research by finding no significant association between work hours and burnout, suggesting work hours alone may not explain high burnout levels in residency.

Although work hours were not directly associated with burnout, both average hours worked and hours worked in the last week were associated with stress levels with effect sizes in the small-to-moderate range.^31^ This aligns with previous studies showing that work hours and stress are linked.^32,33^ The pattern of results suggest that stress had a larger effect size for the associated with hours worked in the last week (β = 0.27 [95% CI, 0.18 to 0.36]) than with average hours worked (β = 0.17 [95% CI, 0.07 to 0.26]). This fits the understanding that stress is situational and in response to life events,^18^ and recent work hours may be more salient than average hours on current stress levels.

While work hours alone may not explain burnout, the association of work hours with elevated stress remains important as prolonged exposure to overwhelming stress leads to burnout.^34^ Although an exploratory finding that did not survive FDR correction, our observation that resilience may attenuate the association between work hours and stress aligns with prior evidence that resilience can help physicians cope with work stressors.^35^ Thus, addressing stress through work hour management and resilience training may ultimately contribute to preventing burnout, even if burnout and work hours are not directly linked. Importantly, while resilience training may help mitigate avoidable distress, relying on resilience training alone risks putting the onus on the individual, and must be coupled with systemic, sustainable change to effectively address resident stress and burnout.^36^

Examination of resident self-assessment of competency milestones offers a contrasting view of work hours, with more hours being associated with higher self-perceived competency. Our finding that a 10-hour increase in average work hours was associated with a 0.11 increase in self-assessed ACGME competency milestones may reflect a meaningful increase in perceived competence given milestones are on a scale of 1 to 5, where level 4 represents readiness to graduate residency.^19^ Notably, effect sizes were in the small-to-moderate range.^31^ This finding adds to the body of evidence that work hour restrictions may have negative consequences on resident education and self-efficacy.^9,37^ However, while there is evidence showing significant alignment between self-evaluation and faculty clinical competency committee milestone scores,^38,39^ our results cannot be interpreted as evidence of program evaluations of resident competency.

Despite investigating a large set of candidate moderators, we found little evidence that demographic or well-being characteristics moderate the association between work hours and outcomes. This suggests that the observed patterns are relatively stable across individuals. We examined specialty as a gross moderator of surgery vs medicine, and there may be more nuanced relationships between specialties that could be explored in future larger studies.

### Limitations

This study has several limitations. First, this survey was part of a randomized trial evaluating a well-being training, introducing potential selection bias. This bias may have resulted in either an overly optimistic (eg, individuals with more bandwidth to join a study may have higher well-being) or overly pessimistic portrayal (eg, individuals seeking to improve their well-being) sample in regard to well-being. However, how these potential biases may influence the relationship between work hours and the outcomes, which was the focus of this paper, is unknown. Second, the majority of participants identified as White women, which may limit generalizability. The racial and ethnic demographics were somewhat similar to ACGME data in the specialties studied,^29^ and the underrepresentation of men is similar to other well-being studies.^40^ Third, due to electronic and snowball recruitment methods, an accurate response rate could not be calculated, which also potentially limits generalizability. While not a probability sample (with a conservative response rate estimate of 0.64% if all eligible residents in the US were reached), the recruitment methods allowed for a broader reach across institutions and demographic groups, contributing to a sample with demographic and training characteristics consistent with national ACGME distributions of US residents.^29^ It is unknown whether the sampling approach affected the primary aim of this study—the association of work hours with the outcomes assessed. Fourth, all measures were self-reported, potentially introducing response or recall bias, though validated scales were used. Program evaluation data of competency milestones and work hours were not used to preserve anonymity and facilitate participation, and work hours are typically self-reported to programs. Furthermore, the measures used were global assessments of well-being-related outcomes, and further research is necessary to explore more immediate, experience-based factors that reflect residents’ current day-to-day realities in relation to work hours. Finally, the cross-sectional design limits our ability to make causal inferences regarding the link between work hours and outcomes. Although it may be more likely that work hours influence the outcomes assessed, we cannot rule out the possibility that the causal direction is reversed.

Our findings have important implications for work hours and well-being in graduate medical education. Our findings challenge the notion that further restricting work hours alone will reduce burnout^11^ and suggest further restrictions could negatively impact residents’ perceived competency. Our results should not be used to support arguments to eliminate work-hour restrictions, either. Rather, these findings indicate that further limiting work hours to prevent burnout is an overly simplistic proposal. Well-being is not simply decreasing negative factors; it is doing so while not affecting the positive indicators such as self-efficacy and personal accomplishment.

Resident burnout is influenced by more than just number of hours worked—it depends on what occurs during work hours, life outside of work, an individual’s relationship to work, and the organizational culture and broader systems within which residents work.^1,2,12^ Furthermore, residency training has evolved alongside shifts in the medical field, including policy, technological, and workforce demographic changes.^41^ What current residents do during work hours and how residents relate to work-life experiences often differs from the training experiences of those training them and the leaders shaping graduate medical education policy. Therefore, a more comprehensive approach that targets the multifaceted contributors to burnout given present-day work circumstances is warranted. Further research is necessary to explore the key drivers of resident burnout and well-being in today’s environment and to identify interventions that support both resident well-being and competency development.

## Conclusions

This nationwide study of resident physicians in high-burnout specialties highlights the varied relationship between work hours, stress, burnout, and self-perceived competency. While longer work hours were associated with higher stress and more self-perceived competency, work hours were not linked to burnout. These findings suggest that work hours alone may not explain high burnout levels in residency. A more comprehensive approach beyond work hour restrictions is needed to support resident well-being in training.

## Notes

Supplement 1. eTable 1. Multilinear regression analysis of work hours and burnout moderators eTable 2. Multilinear regression analysis of work hours and personal accomplishment moderators eTable 3. Multilinear regression analysis of work hours and perceived stress moderators eTable 4. Multilinear regression analysis of work hours and self-assessed milestones moderators eTable 5. Multivariate regression analysis of work hours and burnout moderators eTable 6. Multivariate regression analysis of work hours and personal accomplishment moderators eTable 7. Multivariate regression analysis of work hours and perceived stress moderators eTable 8. Multivariate regression analysis of work hours and self-assessed milestones moderators eTable 9. Variance inflation factor (VIF) values for predictor variables eTable 10. Sensitivity analysis of association of work hours and burnout, personal accomplishment, stress, and milestones eFigure 1. Resilience as a moderator of work hours and stress relationship eFigure 2. Sleep disturbance as a moderator of work hours and milestones relationship eFigure 3. Race as a moderator of work hours and milestones relationship eAppendix. Survey instruments

Supplement 2. Data Sharing Statement
