# Supplementary material for: Work Hours, Stress, and Burnout Among Resident Physicians
Source: JAMA Netw Open. 2026 Jan 14;9(1):e2553974. doi: 10.1001/jamanetworkopen.2025.53974 (PMC12805447; doi:10.1001/jamanetworkopen.2025.53974)
Supplement: Supplement 1. — eTable 1. Multilinear regression analysis of work hours and burnout moderators eTable 2. Multilinear regression analysis of work hours and personal accomplishment moderators eTable 3. Multilinear regression analysis of work hours and perceived stress moderators eTable 4. Multilinear regression analysis of work hours and self-assessed milestones moderators eTable 5. Multivariate regression analysis of work hours and burnout moderators eTable 6. Multivariate regression analysis of work hours and personal accomplishment moderators eTable 7. Multivariate regression analysis of work hours and perceived stress moderators eTable 8. Multivariate regression analysis of work hours and self-assessed milestones moderators eTable 9. Variance inflation factor (VIF) values for predictor variables eTable 10. Sensitivity analysis of association of work hours and burnout, personal accomplishment, stress, and milestones eFigure 1. Resilience as a moderator of work hours and stress relationship eFigure 2. Sleep disturbance as a moderator of work hours and milestones relationship eFigure 3. Race as a moderator of work hours and milestones relationship eAppendix. Survey instruments [file jamanetwopen-e2553974-s001.pdf]

Supplemental Online Content

Tan SF, Siddiqui H, Pinto A, et al. Work hours, stress, and burnout among resident physicians. *JAMA Netw Open.* 2026;9(1):e2553974. doi:10.1001/jamanetworkopen.2025.53974

eTable 1. Multilinear regression analysis of work hours and burnout moderators

eTable 2. Multilinear regression analysis of work hours and personal accomplishment moderators

eTable 3. Multilinear regression analysis of work hours and perceived stress moderators

eTable 4. Multilinear regression analysis of work hours and self-assessed milestones moderators

eTable 5. Multivariate regression analysis of work hours and burnout moderators

eTable 6. Multivariate regression analysis of work hours and personal accomplishment moderators

eTable 7. Multivariate regression analysis of work hours and perceived stress moderators

eTable 8. Multivariate regression analysis of work hours and self-assessed milestones moderators

eTable 9. Variance inflation factor (VIF) values for predictor variables

eTable 10. Sensitivity analysis of association of work hours and burnout, personal accomplishment, stress, and milestones

eFigure 1. Resilience as a moderator of work hours and stress relationship

eFigure 2. Sleep disturbance as a moderator of work hours and milestones relationship

eFigure 3. Race as a moderator of work hours and milestones relationship

eAppendix. Survey instruments

This supplemental material has been provided by the authors to give readers additional information about their work.

© 2025 Tan SF et al. *JAMA Network Open*.

eTable 1: Multilinear Regression Analysis of Work Hours and Burnout[a] Moderators[b]

Variable	Average Work Hours			Work Hours Last Week		
	Estimate, b (95% CI)	p-value	p-adjusted[c]	Estimate, b (95% CI)	p-value	p-adjusted[c]
**Well-being Moderators**						
Resilience[d] x Work Hours	-0.04 (-0.10, 0.02)	0.196	0.987	-0.02 (-0.06, 0.02)	0.403	0.987
Flourishing[e] x Work Hours	0.00 (-0.03, 0.03)	0.863	0.987	0.00 (-0.02, 0.02)	0.822	0.987
Mindful Acting Awareness[f] x Work Hours	-0.02 (-0.08, 0.04)	0.613	0.987	-0.04 (-0.08, 0.00)	0.076	0.987
Mindful Non-Judgement[g] x Work Hours	0.00 (-0.05, 0.05)	0.968	0.987	0.01 (-0.02, 0.05)	0.536	0.987
Loneliness[h] x Work Hours	0.01 (-0.04, 0.06)	0.693	0.987	0.01 (-0.03, 0.04)	0.760	0.987
Meaning and Purpose[i] x Work Hours	0.00 (0.00, 0.01)	0.972	0.987	0.00 (0.00, 0.00)	0.766	0.987
Sleep Disturbance[j] x Work Hours	0.00 (-0.01, 0.00)	0.328	0.987	0.00 (0.00, 0.00)	0.713	0.987
**Demographic Moderators**						
Gender[k]: Cisgender Man x Work Hours	-0.01 (-0.11, 0.09)	0.840	0.987	-0.01 (-0.09, 0.06)	0.747	0.987
Gender: Transgender/Nonbinary x Work Hours	-0.01 (-0.53, 0.51)	0.984	0.987	0.00 (-0.27, 0.28)	0.986	0.987
Race[l]: Asian x Work Hours	-0.12 (-0.25, 0.01)	0.063	0.987	-0.04 (-0.13, 0.04)	0.331	0.987
Race: Black x Work Hours	0.07 (-0.13, 0.27)	0.484	0.987	0.06 (-0.10, 0.21)	0.462	0.987
Race: Multiracial/Other x Work Hours	0.10 (-0.11, 0.31)	0.337	0.987	0.04 (-0.10, 0.17)	0.577	0.987
Ethnicity[m]: Hispanic x Work Hours	0.04 (-0.14, 0.22)	0.659	0.987	0.00 (-0.14, 0.14)	0.987	0.987
Specialty[n]: Surgery x Work Hours	0.11 (-0.01, 0.23)	0.061	0.987	0.04 (-0.04, 0.11)	0.313	0.987
Training Level[o]: Senior x Work Hours	-0.03 (-0.17, 0.11)	0.665	0.987	0.00 (-0.09, 0.10)	0.939	0.987
Training Level: Mid-Level x Work Hours	-0.05 (-0.17, 0.07)	0.416	0.987	-0.05 (-0.12, 0.03)	0.229	0.987

a. Abbreviated Maslach Burnout Inventory-Human Services Survey (MBI-HSS) Emotional Exhaustion and Depersonalization subscale range: 0-36. Higher scores indicate higher burnout.
b. Multiple linear regression models of work hours predicting burnout while controlling for demographics (gender, race, ethnicity, specialty, training level, relationship statues, geography, and season of survey completion) and other well-being moderators post Variance Inflation Factor (VIF) Adjustment. Each model fit one interaction of interest per model. In the full models without interaction terms, the variable Flourishing had a VIF score of approximately 3.3. While VIF values above 10 are generally considered problematic, some statisticians (e.g. Paul Allison) suggest caution for values above 2.5. To ensure robustness, all full models with interaction terms were re-estimated after removing Flourishing. For models testing interactions involving Flourishing, the covariate Meaning and Purpose was removed, which reduced the VIF for Flourishing to 2.4.
c. Benjamini-Hochberg False-Discover Rate adjusted p-values.
d. Brief Resilience Scale range: 0-6, higher scores indicating higher resilience.
e. Flourishing Index range: 0-10, higher scores indicating higher flourishing.
f. Five Facet Mindfulness Questionnaire Acting with Awareness Subscale range: 1-5, higher scores indicate a greater ability to act with awareness.
g. Five Facet Mindfulness Questionnaire Non-Judgement Subscale range: 1-5, higher scores indicates greater non-judgement.
h. NIH Toolbox Loneliness range: 1-5, higher scores indicate a greater sense of loneliness.
i. PROMIS Meaning and Purpose scored as T-scores (mean = 50, SD = 10 where higher scores indicate more meaning and purpose in life.
j. PROMIS Sleep Disturbance Scale scored as T-scores (mean = 50, SD = 10) where higher scores indicate more sleep disturbance.
k. Gender reference group: Cisgendered Woman
l. Race reference group: White
m. Ethnicity reference group: Non-Hispanic
n. Specialty reference group: Medical Specialty
o. Training Level reference group: Intern/PGY1

© 2025 Tan SF et al. *JAMA Network Open*.

eTable 2: Multilinear Regression Analysis of Work Hours and Personal Accomplishment[a] Moderators[b]

Variable	Average Work Hours			Work Hours Last Week		
	Estimate, b (95% CI)	p-value	p-adjusted[c]	Estimate, b (95% CI)	p-value	p-adjusted[c]
**Well-being Moderators**						
Resilience[d] x Work Hours	0.01 (-0.02, 0.03)	0.575	0.939	0.01 (-0.01, 0.02)	0.514	0.939
Flourishing[e] x Work Hours	0.00 (-0.01, 0.01)	0.756	0.939	0.00 (-0.01, 0.01)	0.787	0.939
Mindful Acting Awareness[f] x Work Hours	0.01 (-0.02, 0.03)	0.524	0.939	0.01 (-0.01, 0.03)	0.240	0.939
Mindful Non-Judgement[g] x Work Hours	-0.01 (-0.03, 0.02)	0.601	0.939	0.00 (-0.01, 0.01)	0.913	0.939
Loneliness[h] x Work Hours	-0.01 (-0.03, 0.01)	0.274	0.939	-0.01 (-0.02, 0.01)	0.386	0.939
Meaning and Purpose[i] x Work Hours	0.00 (0.00, 0.00)	0.190	0.939	0.00 (0.00, 0.00)	0.586	0.939
Sleep Disturbance[j] x Work Hours	0.00 (0.00, 0.00)	0.939	0.939	0.00 (0.00, 0.00)	0.602	0.939
**Demographic Moderators**						
Gender[k]: Cisgender Man x Work Hours	0.03 (-0.01, 0.07)	0.180	0.939	0.02 (-0.01, 0.05)	0.159	0.939
Gender: Transgender/Nonbinary x Work Hours	0.14 (-0.06, 0.33)	0.179	0.939	0.03 (-0.07, 0.14)	0.541	0.939
Race[l]: Asian x Work Hours	-0.04 (-0.09, 0.01)	0.106	0.939	-0.01 (-0.04, 0.03)	0.741	0.939
Race: Black x Work Hours	0.02 (-0.06, 0.09)	0.616	0.939	0.03 (-0.03, 0.09)	0.387	0.939
Race: Multiracial/Other x Work Hours	-0.05 (-0.13, 0.03)	0.204	0.939	-0.01 (-0.06, 0.04)	0.627	0.939
Ethnicity[m]: Hispanic x Work Hours	0.00 (-0.07, 0.06)	0.890	0.939	0.02 (-0.04, 0.07)	0.552	0.939
Specialty[n]: Surgery x Work Hours	0.02 (-0.02, 0.07)	0.295	0.939	0.02 (-0.01, 0.05)	0.161	0.939
Training Level[o]: Senior x Work Hours	0.01 (-0.05, 0.06)	0.834	0.939	-0.02 (-0.06, 0.01)	0.207	0.939
Training Level: Mid-Level x Work Hours	0.01 (-0.04, 0.06)	0.733	0.939	-0.01 (-0.04, 0.02)	0.495	0.939

a. Abbreviated Maslach Burnout Inventory-Human Services Survey (MBI-HSS) Personal Accomplishment subscale range: 0-18. Higher scores indicate higher personal accomplishment and lower burnout.
b. Multiple linear regression models of work hours predicting personal accomplishment while controlling for demographics (gender, race, ethnicity, specialty, training level, relationship statues, geography, and season of survey completion) and other well-being moderators post Variance Inflation Factor (VIF) Adjustment. In the full models without interaction terms, the variable Flourishing had a VIF score of approximately 3.3. Each model fit one interaction of interest per model. While VIF values above 10 are generally considered problematic, some statisticians (e.g. Paul Allison) suggest caution for values above 2.5. To ensure robustness, all full models with interaction terms were re-estimated after removing Flourishing. For models testing interactions involving Flourishing, the covariate Meaning and Purpose was removed, which reduced the VIF for Flourishing to 2.4.
c. Benjamini-Hochberg False-Discover Rate adjusted p-values.
d. Brief Resilience Scale range: 0-6, higher scores indicating higher resilience.
e. Flourishing Index range: 0-10, higher scores indicating higher flourishing.
f. Five Facet Mindfulness Questionnaire Acting with Awareness Subscale range: 1-5, higher scores indicate a greater ability to act with awareness.
g. Five Facet Mindfulness Questionnaire Non-Judgement Subscale range: 1-5, higher scores indicates greater non-judgement.
h. NIH Toolbox Loneliness range: 1-5, higher scores indicate a greater sense of loneliness.
i. PROMIS Meaning and Purpose scored as T-scores (mean = 50, SD = 10 where higher scores indicate more meaning and purpose in life.
j. PROMIS Sleep Disturbance Scale scored as T-scores (mean = 50, SD = 10) where higher scores indicate more sleep disturbance.
k. Gender reference group: Cisgendered Woman

© 2025 Tan SF et al. *JAMA Network Open*.

l. Race reference group: White
m. Ethnicity reference group: Non-Hispanic
n. Specialty reference group: Medical Specialty
o. Training Level reference group: Intern/PGY1

© 2025 Tan SF et al. *JAMA Network Open*.

eTable 3: Multilinear Regression Analysis of Work Hours and Perceived Stress[a] Moderators[b]

Variable	Average Work Hours			Work Hours Last Week		
	Estimate, b (95% CI)	p-value	p-adjusted[c]	Estimate, b (95% CI)	p-value	p-adjusted[c]
**Well-being Moderators**						
Resilience[d] x Work Hours	-0.06 (-0.11, -0.01)	0.011	0.344	-0.03 (-0.05, 0.00)	0.079	0.996
Flourishing[e] x Work Hours	-0.01 (-0.03, 0.02)	0.590	0.996	0.00 (-0.02, 0.01)	0.745	0.996
Mindful Acting Awareness[f] x Work Hours	-0.02 (-0.07, 0.02)	0.327	0.996	0.00 (-0.03, 0.03)	0.897	0.996
Mindful Non-Judgement[g] x Work Hours	-0.04 (-0.08, 0.00)	0.061	0.996	-0.01 (-0.03, 0.02)	0.574	0.996
Loneliness[h] x Work Hours	0.02 (-0.02, 0.06)	0.252	0.996	0.01 (-0.01, 0.04)	0.319	0.996
Meaning and Purpose[i] x Work Hours	0.00 (-0.01, 0.00)	0.139	0.996	0.00 (0.00, 0.00)	0.816	0.996
Sleep Disturbance[j] x Work Hours	0.00 (0.00, 0.01)	0.102	0.996	0.00 (0.00, 0.00)	0.511	0.996
**Demographic Moderators**						
Gender[k]: Cisgender Man x Work Hours	-0.01 (-0.08, 0.07)	0.853	0.996	-0.01 (-0.06, 0.04)	0.741	0.996
Gender: Transgender/Nonbinary x Work Hours	-0.06 (-0.44, 0.33)	0.773	0.996	-0.08 (-0.28, 0.11)	0.408	0.996
Race[l]: Asian x Work Hours	0.07 (-0.03, 0.16)	0.180	0.996	-0.01 (-0.07, 0.05)	0.844	0.996
Race: Black x Work Hours	-0.02 (-0.16, 0.13)	0.837	0.996	0.10 (-0.01, 0.21)	0.067	0.996
Race: Multiracial/Other x Work Hours	0.12 (-0.04, 0.27)	0.136	0.996	0.00 (-0.10, 0.10)	0.996	0.996
Ethnicity[m]: Hispanic x Work Hours	0.04 (-0.09, 0.17)	0.541	0.996	-0.06 (-0.16, 0.04)	0.242	0.996
Specialty[n]: Surgery x Work Hours	0.05 (-0.04, 0.13)	0.305	0.996	0.03 (-0.02, 0.08)	0.234	0.996
Training Level[o]: Senior x Work Hours	0.08 (-0.02, 0.18)	0.122	0.996	0.06 (-0.01, 0.13)	0.073	0.996
Training Level: Mid-Level x Work Hours	0.07 (-0.02, 0.16)	0.134	0.996	0.02 (-0.04, 0.07)	0.580	0.996

a. Perceived Stress Scale (PSS) range: 0-40, higher scores indicate greater perceived stress.
b. Multiple linear regression models of work hours predicting stress while controlling for demographics (gender, race, ethnicity, specialty, training level, relationship statues, geography, and season of survey completion) and other well-being moderators post Variance Inflation Factor (VIF) Adjustment. Each model fit one interaction of interest per model. In the full models without interaction terms, the variable Flourishing had a VIF score of approximately 3.3. While VIF values above 10 are generally considered problematic, some statisticians (e.g. Paul Allison) suggest caution for values above 2.5. To ensure robustness, all full models with interaction terms were re-estimated after removing Flourishing. For models testing interactions involving Flourishing, the covariate Meaning and Purpose was removed, which reduced the VIF for Flourishing to 2.4.
c. Benjamini-Hochberg False-Discover Rate adjusted p-values.
d. Brief Resilience Scale range: 0-6, higher scores indicating higher resilience.
e. Flourishing Index range: 0-10, higher scores indicating higher flourishing.
f. Five Facet Mindfulness Questionnaire Acting with Awareness Subscale range: 1-5, higher scores indicate a greater ability to act with awareness.
g. Five Facet Mindfulness Questionnaire Non-Judgement Subscale range: 1-5, higher scores indicates greater non-judgement.
h. NIH Toolbox Loneliness range: 1-5, higher scores indicate a greater sense of loneliness.
i. PROMIS Meaning and Purpose scored as T-scores (mean = 50, SD = 10 where higher scores indicate more meaning and purpose in life.
j. PROMIS Sleep Disturbance Scale scored as T-scores (mean = 50, SD = 10) where higher scores indicate more sleep disturbance.
k. Gender reference group: Cisgendered Woman
l. Race reference group: White
m. Ethnicity reference group: Non-Hispanic
n. Specialty reference group: Medical Specialty
o. Training Level reference group: Intern/PGY1

© 2025 Tan SF et al. *JAMA Network Open*.

eTable 4: Multilinear Regression Analysis of Work Hours and Self-Assessed Milestones[a] Moderators[b]

Variable	Average Work Hours			Work Hours Last Week		
	Estimate, b (95% CI)	p-value	p-adjusted[c]	Estimate, b (95% CI)	p-value	p-adjusted[c]
**Well-being Moderators**						
Resilience[d] x Work Hours	-0.01 (-0.01, 0.00)	0.065	0.980	0.00 (0.00, 0.00)	0.796	0.980
Flourishing[e] x Work Hours	0.00 (0.00, 0.00)	0.575	0.980	0.00 (0.00, 0.00)	0.501	0.980
Mindful Acting Awareness[f] x Work Hours	0.00 (-0.01, 0.00)	0.205	0.980	0.00 (-0.01, 0.00)	0.122	0.980
Mindful Non-Judgement[g] x Work Hours	0.00 (-0.01, 0.00)	0.339	0.980	0.00 (0.00, 0.00)	0.605	0.980
Loneliness[h] x Work Hours	0.00 (0.00, 0.01)	0.745	0.980	0.00 (0.00, 0.01)	0.138	0.980
Meaning and Purpose[i] x Work Hours	0.00 (0.00, 0.00)	0.682	0.980	0.00 (0.00, 0.00)	0.723	0.980
Sleep Disturbance[j] x Work Hours	0.00 (0.00, 0.00)	0.912	0.980	0.00 (0.00, 0.00)	0.036	0.980
**Demographic Moderators**						
Gender[k]: Cisgender Man x Work Hours	0.00 (-0.01, 0.01)	0.644	0.980	0.01 (0.00, 0.01)	0.083	0.980
Gender: Transgender/Nonbinary x Work Hours	-0.01 (-0.07, 0.04)	0.607	0.980	0.00 (-0.03, 0.03)	0.980	0.980
Race[l]: Asian x Work Hours	-0.01 (-0.03, 0.00)	0.080	0.980	-0.01 (-0.02, 0.00)	0.025	0.813
Race: Black x Work Hours	-0.01 (-0.03, 0.01)	0.544	0.980	0.00 (-0.02, 0.02)	0.959	0.980
Race: Multiracial/Other x Work Hours	0.00 (-0.02, 0.03)	0.687	0.980	0.01 (0.00, 0.03)	0.081	0.980
Ethnicity[m]: Hispanic x Work Hours	0.01 (-0.01, 0.03)	0.396	0.980	0.00 (-0.01, 0.02)	0.530	0.980
Specialty[n]: Surgery x Work Hours	-0.01 (-0.02, 0.01)	0.367	0.980	0.01 (0.00, 0.01)	0.075	0.980
Training Level[o]: Senior x Work Hours	0.00 (-0.01, 0.02)	0.497	0.980	0.01 (0.00, 0.02)	0.141	0.980
Training Level: Mid-Level x Work Hours	-0.01 (-0.02, 0.00)	0.163	0.980	0.00 (-0.01, 0.01)	0.491	0.980

a. The Accreditation Council for Graduate Medical Education Milestones range: 1-5, higher scores indicate higher self-assessment of core competencies in Patient Care, Practice-Based Learning and Improvement, Professionalism, and Interpersonal and Communication Skills.
b. Multiple linear regression models of work hours predicting self-assessed ACGME competency milestones while controlling for demographics (gender, race, ethnicity, specialty, training level, relationship statues, geography, and season of survey completion) and other well-being moderators post Variance Inflation Factor (VIF) Adjustment. Each model fit one interaction of interest per model. In the full models without interaction terms, the variable Flourishing had a VIF score of approximately 3.3. While VIF values above 10 are generally considered problematic, some statisticians (e.g. Paul Allison) suggest caution for values above 2.5. To ensure robustness, all full models with interaction terms were re-estimated after removing Flourishing. For models testing interactions involving Flourishing, the covariate Meaning and Purpose was removed, which reduced the VIF for Flourishing to 2.4.
c. Benjamini-Hochberg False-Discover Rate adjusted p-values.
d. Brief Resilience Scale range: 0-6, higher scores indicating higher resilience.
e. Flourishing Index range: 0-10, higher scores indicating higher flourishing.
f. Five Facet Mindfulness Questionnaire Acting with Awareness Subscale range: 1-5, higher scores indicate a greater ability to act with awareness.
g. Five Facet Mindfulness Questionnaire Non-Judgement Subscale range: 1-5, higher scores indicates greater non-judgement.
h. NIH Toolbox Loneliness range: 1-5, higher scores indicate a greater sense of loneliness.
i. PROMIS Meaning and Purpose scored as T-scores (mean = 50, SD = 10 where higher scores indicate more meaning and purpose in life.
j. PROMIS Sleep Disturbance Scale scored as T-scores (mean = 50, SD = 10) where higher scores indicate more sleep disturbance.

© 2025 Tan SF et al. *JAMA Network Open*.

k. Gender reference group: Cisgendered Woman
l. Race reference group: White
m. Ethnicity reference group: Non-Hispanic
n. Specialty reference group: Medical Specialty
o. Training Level reference group: Intern/PGY1

© 2025 Tan SF et al. *JAMA Network Open*.

eTable 5: Multivariate Regression Analysis of Work Hours and Burnout[a] Moderators[b]

Variable	Average Work Hours			Work Hours Last Week		
	Estimate, b (95% CI)	p-value	p-adjusted[c]	Estimate, b (95% CI)	p-value	p-adjusted[c]
**Well-being Moderators**						
Resilience[d] x Work Hours	-0.09 (-0.18, -0.01)	0.034	0.256	-0.03 (-0.09, 0.02)	0.219	0.668
Mindful Acting Awareness[f] x Work Hours	-0.01 (-0.09, 0.07)	0.850	0.865	-0.06 (-0.11, -0.00)	0.034	0.512
Mindful Non-Judgement[g] x Work Hours	0.01 (-0.06, 0.07)	0.865	0.865	0.03 (-0.01, 0.07)	0.189	0.668
Loneliness[h] x Work Hours	0.01 (-0.07, 0.09)	0.812	0.865	-0.01 (-0.06, 0.04)	0.736	0.998
Meaning and Purpose[i] x Work Hours	-0.00 (-0.01, 0.01)	0.834	0.865	0.00 (-0.00, 0.00)	0.913	0.998
Sleep Disturbance[j] x Work Hours	-0.01 (-0.02, 0.00)	0.077	0.384	-0.00 (-0.01, 0.00)	0.312	0.668
**Demographic Moderators**						
Gender[k]: Cisgender Man x Work Hours	0.02 (-0.10, 0.14)	0.742	0.865	-0.01 (-0.09, 0.07)	0.850	0.998
Gender: Transgender/Nonbinary x Work Hours	-0.05 (-0.57, 0.48)	0.857	0.865	-0.00 (-0.28, 0.28)	0.992	0.998
Race[l]: Asian x Work Hours	-0.11 (-0.25, 0.02)	0.103	0.384	-0.03 (-0.12, 0.05)	0.465	0.872
Race: Black x Work Hours	0.07 (-0.14, 0.29)	0.513	0.865	0.09 (-0.07, 0.25)	0.268	0.668
Race: Multiracial/Other x Work Hours	0.10 (-0.12, 0.32)	0.383	0.837	-0.01 (-0.15, 0.14)	0.933	0.998
Ethnicity[m]: Hispanic x Work Hours	0.05 (-0.16, 0.25)	0.661	0.865	0.00 (-0.15, 0.15)	0.998	0.998
Specialty[n]: Surgery x Work Hours	0.14 (0.02, 0.27)	0.026	0.256	0.05 (-0.03, 0.13)	0.192	0.668
Training Level[o]: Senior x Work Hours	-0.06 (-0.21, 0.08)	0.391	0.837	0.02 (-0.08, 0.11)	0.724	0.998
Training Level: Mid-Level x Work Hours	-0.10 (-0.23, 0.04)	0.166	0.499	-0.04 (-0.13, 0.04)	0.295	0.668

a. Abbreviated Maslach Burnout Inventory-Human Services Survey (MBI-HSS) Emotional Exhaustion and Depersonalization subscale range: 0-36. Higher scores indicate higher burnout.
b. Multivariate linear regression models were used to examine the relationship between work hours and burnout, adjusting for a comprehensive set of demographic covariates (gender, race, ethnicity, specialty, training level, relationship status, geographic region, and season of survey completion) as well as other well-being moderators. Each model for average work hours and work hours last week includes all moderators and interactions. Following assessment of multicollinearity using Variance Inflation Factor (VIF), the variable Flourishing was excluded from all full models with interaction terms due to a VIF of approximately 3.3. Although VIF values above 10 are typically considered problematic, some statisticians (e.g., Paul Allison) recommend caution for values exceeding 2.5.
c. Benjamini-Hochberg False-Discover Rate adjusted p-values.
d. Brief Resilience Scale range: 0-6, higher scores indicating higher resilience.
e. Flourishing Index range: 0-10, higher scores indicating higher flourishing.
f. Five Facet Mindfulness Questionnaire Acting with Awareness Subscale range: 1-5, higher scores indicate a greater ability to act with awareness.
g. Five Facet Mindfulness Questionnaire Non-Judgement Subscale range: 1-5, higher scores indicates greater non-judgement.
h. NIH Toolbox Loneliness range: 1-5, higher scores indicate a greater sense of loneliness.
i. PROMIS Meaning and Purpose scored as T-scores (mean = 50, SD = 10 where higher scores indicate more meaning and purpose in life.
j. PROMIS Sleep Disturbance Scale scored as T-scores (mean = 50, SD = 10) where higher scores indicate more sleep disturbance.
k. Gender reference group: Cisgendered Woman
l. Race reference group: White
m. Ethnicity reference group: Non-Hispanic
n. Specialty reference group: Medical Specialty
o. Training Level reference group: Intern/PGY1

© 2025 Tan SF et al. *JAMA Network Open*.

eTable 6: Multivariate Regression Analysis of Work Hours and Personal Accomplishment[a] Moderators[b]

Variable	Average Work Hours			Work Hours Last Week		
	Estimate, b (95% CI)	p-value	p-adjusted[c]	Estimate, b (95% CI)	p-value	p-adjusted[c]
**Well-being Moderators**						
Resilience[d] x Work Hours	-0.00 (-0.03, 0.03)	0.935	0.935	-0.00 (-0.02, 0.02)	0.791	0.912
Mindful Acting Awareness[f] x Work Hours	0.01 (-0.03, 0.04)	0.713	0.822	0.01 (-0.01, 0.03)	0.530	0.912
Mindful Non-Judgement[g] x Work Hours	-0.02 (-0.04, 0.01)	0.250	0.774	-0.00 (-0.02, 0.01)	0.692	0.912
Loneliness[h] x Work Hours	-0.01 (-0.04, 0.02)	0.453	0.774	-0.00 (-0.02, 0.02)	0.717	0.912
Meaning and Purpose[i] x Work Hours	0.00 (-0.00, 0.00)	0.322	0.774	-0.00 (-0.00, 0.00)	0.756	0.912
Sleep Disturbance[j] x Work Hours	0.00 (-0.00, 0.00)	0.225	0.774	0.00 (-0.00, 0.00)	0.936	0.969
**Demographic Moderators**						
Gender[k]: Cisgender Man x Work Hours	0.02 (-0.03, 0.07)	0.393	0.774	0.02 (-0.01, 0.05)	0.187	0.912
Gender: Transgender/Nonbinary x Work Hours	0.15 (-0.05, 0.36)	0.134	0.774	0.03 (-0.08, 0.14)	0.613	0.912
Race[l]: Asian x Work Hours	-0.04 (-0.09, 0.01)	0.126	0.774	-0.00 (-0.03, 0.03)	0.969	0.969
Race: Black x Work Hours	0.02 (-0.07, 0.10)	0.679	0.822	0.03 (-0.03, 0.09)	0.313	0.912
Race: Multiracial/Other x Work Hours	-0.04 (-0.12, 0.05)	0.370	0.774	-0.01 (-0.07, 0.04)	0.627	0.912
Ethnicity[m]: Hispanic x Work Hours	-0.02 (-0.10, 0.06)	0.612	0.822	0.02 (-0.04, 0.08)	0.493	0.912
Specialty[n]: Surgery x Work Hours	0.02 (-0.03, 0.07)	0.465	0.774	0.02 (-0.00, 0.05)	0.103	0.912
Training Level[o]: Senior x Work Hours	-0.01 (-0.07, 0.04)	0.645	0.822	-0.02 (-0.06, 0.01)	0.229	0.912
Training Level: Mid-Level x Work Hours	0.00 (-0.05, 0.06)	0.852	0.913	-0.01 (-0.04, 0.02)	0.635	0.912

a. Abbreviated Maslach Burnout Inventory-Human Services Survey (MBI-HSS) Personal Accomplishment subscale range: 0-18. Higher scores indicate higher personal accomplishment and lower burnout.
b. Multivariate linear regression models were used to examine the relationship between work hours and personal accomplishment, adjusting for a comprehensive set of demographic covariates (gender, race, ethnicity, specialty, training level, relationship status, geographic region, and season of survey completion) as well as other well-being moderators. Each model for average work hours and work hours last week includes all moderators and interactions. Following assessment of multicollinearity using Variance Inflation Factor (VIF), the variable Flourishing was excluded from all full models with interaction terms due to a VIF of approximately 3.3. Although VIF values above 10 are typically considered problematic, some statisticians (e.g., Paul Allison) recommend caution for values exceeding 2.5.
c. Benjamini-Hochberg False-Discover Rate adjusted p-values.
d. Brief Resilience Scale range: 0-6, higher scores indicating higher resilience.
e. Flourishing Index range: 0-10, higher scores indicating higher flourishing.
f. Five Facet Mindfulness Questionnaire Acting with Awareness Subscale range: 1-5, higher scores indicate a greater ability to act with awareness.
g. Five Facet Mindfulness Questionnaire Non-Judgement Subscale range: 1-5, higher scores indicates greater non-judgement.
h. NIH Toolbox Loneliness range: 1-5, higher scores indicate a greater sense of loneliness.
i. PROMIS Meaning and Purpose scored as T-scores (mean = 50, SD = 10 where higher scores indicate more meaning and purpose in life.
j. PROMIS Sleep Disturbance Scale scored as T-scores (mean = 50, SD = 10) where higher scores indicate more sleep disturbance.
k. Gender reference group: Cisgendered Woman
l. Race reference group: White
m. Ethnicity reference group: Non-Hispanic
n. Specialty reference group: Medical Specialty
o. Training Level reference group: Intern/PGY1

© 2025 Tan SF et al. *JAMA Network Open*.

eTable 7: Multivariate Regression Analysis of Work Hours and Perceived Stress[a] Moderators[b]

Variable	Average Work Hours			Work Hours Last Week		
	Estimate, b (95% CI)	p-value	p-adjusted[c]	Estimate, b (95% CI)	p-value	p-adjusted[c]
**Well-being Moderators**						
Resilience[d] x Work Hours	-0.08 (-0.14, -0.01)	0.020	0.295	-0.04 (-0.08, -0.00)	0.029	0.195
Mindful Acting Awareness[f] x Work Hours	0.03 (-0.03, 0.08)	0.410	0.684	0.02 (-0.02, 0.06)	0.336	0.839
Mindful Non-Judgement[g] x Work Hours	-0.03 (-0.07, 0.02)	0.322	0.603	0.00 (-0.03, 0.03)	0.914	0.979
Loneliness[h] x Work Hours	-0.01 (-0.07, 0.05)	0.766	0.911	0.00 (-0.04, 0.04)	0.910	0.979
Meaning and Purpose[i] x Work Hours	-0.00 (-0.01, 0.01)	0.850	0.911	0.00 (-0.00, 0.00)	0.979	0.979
Sleep Disturbance[j] x Work Hours	0.00 (-0.01, 0.01)	0.938	0.938	0.00 (-0.00, 0.00)	0.935	0.979
**Demographic Moderators**						
Gender[k]: Cisgender Man x Work Hours	0.02 (-0.07, 0.11)	0.682	0.911	0.01 (-0.05, 0.07)	0.780	0.979
Gender: Transgender/Nonbinary x Work Hours	-0.04 (-0.43, 0.35)	0.838	0.911	-0.05 (-0.25, 0.15)	0.631	0.979
Race[l]: Asian x Work Hours	0.07 (-0.03, 0.17)	0.157	0.523	-0.00 (-0.06, 0.06)	0.937	0.979
Race: Black x Work Hours	-0.04 (-0.20, 0.12)	0.661	0.911	0.13 (0.02, 0.24)	0.025	0.195
Race: Multiracial/Other x Work Hours	0.11 (-0.05, 0.27)	0.189	0.523	0.02 (-0.09, 0.12)	0.750	0.979
Ethnicity[m]: Hispanic x Work Hours	0.09 (-0.06, 0.24)	0.237	0.523	-0.05 (-0.16, 0.05)	0.330	0.839
Specialty[n]: Surgery x Work Hours	0.06 (-0.03, 0.16)	0.182	0.523	0.04 (-0.01, 0.10)	0.108	0.404
Training Level[o]: Senior x Work Hours	0.09 (-0.02, 0.20)	0.112	0.523	0.07 (0.00, 0.14)	0.039	0.195
Training Level: Mid-Level x Work Hours	0.06 (-0.04, 0.16)	0.244	0.523	0.01 (-0.05, 0.07)	0.813	0.979

a. Perceived Stress Scale (PSS) range: 0-40, higher scores indicate greater perceived stress.
b. Multivariate linear regression models were used to examine the relationship between work hours and stress, adjusting for a comprehensive set of demographic covariates (gender, race, ethnicity, specialty, training level, relationship status, geographic region, and season of survey completion) as well as other well-being moderators. Each model for average work hours and work hours last week includes all moderators and interactions. Following assessment of multicollinearity using Variance Inflation Factor (VIF), the variable Flourishing was excluded from all full models with interaction terms due to a VIF of approximately 3.3. Although VIF values above 10 are typically considered problematic, some statisticians (e.g., Paul Allison) recommend caution for values exceeding 2.5.
c. Benjamini-Hochberg False-Discover Rate adjusted p-values.
d. Brief Resilience Scale range: 0-6, higher scores indicating higher resilience.
e. Flourishing Index range: 0-10, higher scores indicating higher flourishing.
f. Five Facet Mindfulness Questionnaire Acting with Awareness Subscale range: 1-5, higher scores indicate a greater ability to act with awareness.
g. Five Facet Mindfulness Questionnaire Non-Judgement Subscale range: 1-5, higher scores indicates greater non-judgement.
h. NIH Toolbox Loneliness range: 1-5, higher scores indicate a greater sense of loneliness.
i. PROMIS Meaning and Purpose scored as T-scores (mean = 50, SD = 10 where higher scores indicate more meaning and purpose in life.
j. PROMIS Sleep Disturbance Scale scored as T-scores (mean = 50, SD = 10) where higher scores indicate more sleep disturbance.
k. Gender reference group: Cisgendered Woman
l. Race reference group: White
m. Ethnicity reference group: Non-Hispanic
n. Specialty reference group: Medical Specialty

© 2025 Tan SF et al. *JAMA Network Open*.

- **o.** Training Level reference group: Intern/PGY1

© 2025 Tan SF et al. *JAMA Network Open*.

eTable 8: Multivariate Regression Analysis of Work Hours and Self-Assessed Milestones[a] Moderators[b]

Variable	Average Work Hours			Work Hours Last Week		
	Estimate, b (95% CI)	p-value	p-adjusted[c]	Estimate, b (95% CI)	p-value	p-adjusted[c]
**Well-being Moderators**						
Resilience[d] x Work Hours	-0.01 (-0.02, -0.00)	0.040	0.529	-0.00 (-0.01, 0.00)	0.853	0.985
Mindful Acting Awareness[f] x Work Hours	-0.00 (-0.01, 0.01)	0.730	0.759	-0.00 (-0.01, 0.00)	0.605	0.985
Mindful Non-Judgement[g] x Work Hours	-0.00 (-0.01, 0.00)	0.469	0.759	0.00 (-0.00, 0.00)	0.931	0.985
Loneliness[h] x Work Hours	-0.00 (-0.01, 0.01)	0.759	0.759	0.00 (-0.00, 0.01)	0.558	0.985
Meaning and Purpose[i] x Work Hours	-0.00 (-0.00, 0.00)	0.724	0.759	0.00 (-0.00, 0.00)	0.940	0.985
Sleep Disturbance[j] x Work Hours	-0.00 (-0.00, 0.00)	0.347	0.759	0.00 (-0.00, 0.00)	0.197	0.493
**Demographic Moderators**						
Gender[k]: Cisgender Man x Work Hours	0.00 (-0.01, 0.01)	0.718	0.759	0.01 (-0.00, 0.02)	0.096	0.493
Gender: Transgender/Nonbinary x Work Hours	-0.01 (-0.06, 0.04)	0.663	0.759	0.00 (-0.03, 0.03)	0.878	0.985
Race[l]: Asian x Work Hours	-0.01 (-0.03, 0.00)	0.071	0.529	-0.01 (-0.02, 0.00)	0.106	0.493
Race: Black x Work Hours	-0.00 (-0.03, 0.02)	0.747	0.759	0.00 (-0.01, 0.02)	0.573	0.985
Race: Multiracial/Other x Work Hours	0.01 (-0.02, 0.03)	0.656	0.759	0.01 (-0.00, 0.02)	0.191	0.493
Ethnicity[m]: Hispanic x Work Hours	0.00 (-0.02, 0.02)	0.719	0.759	0.00 (-0.02, 0.02)	0.985	0.985
Specialty[n]: Surgery x Work Hours	-0.01 (-0.02, 0.01)	0.438	0.759	0.01 (-0.00, 0.02)	0.056	0.493
Training Level[o]: Senior x Work Hours	0.01 (-0.01, 0.02)	0.497	0.759	0.01 (-0.00, 0.02)	0.191	0.493
Training Level: Mid-Level x Work Hours	-0.01 (-0.02, 0.00)	0.135	0.673	-0.00 (-0.01, 0.01)	0.868	0.985

a. The Accreditation Council for Graduate Medical Education Milestones range: 1-5, higher scores indicate higher self-assessment of core competencies in Patient Care, Practice-Based Learning and Improvement, Professionalism, and Interpersonal and Communication Skills.
b. Multivariate linear regression models were used to examine the relationship between work hours and self-assessed ACGME competency milestones, adjusting for a comprehensive set of demographic covariates (gender, race, ethnicity, specialty, training level, relationship status, geographic region, and season of survey completion) as well as other well-being moderators. Each model for average work hours and work hours last week includes all moderators and interactions. Following assessment of multicollinearity using Variance Inflation Factor (VIF), the variable Flourishing was excluded from all full models with interaction terms due to a VIF of approximately 3.3. Although VIF values above 10 are typically considered problematic, some statisticians (e.g., Paul Allison) recommend caution for values exceeding 2.5.
c. Benjamini-Hochberg False-Discover Rate adjusted p-values.
d. Brief Resilience Scale range: 0-6, higher scores indicating higher resilience.
e. Flourishing Index range: 0-10, higher scores indicating higher flourishing.
f. Five Facet Mindfulness Questionnaire Acting with Awareness Subscale range: 1-5, higher scores indicate a greater ability to act with awareness.
g. Five Facet Mindfulness Questionnaire Non-Judgement Subscale range: 1-5, higher scores indicates greater non-judgement.
h. NIH Toolbox Loneliness range: 1-5, higher scores indicate a greater sense of loneliness.
i. PROMIS Meaning and Purpose scored as T-scores (mean = 50, SD = 10 where higher scores indicate more meaning and purpose in life.
j. PROMIS Sleep Disturbance Scale scored as T-scores (mean = 50, SD = 10) where higher scores indicate more sleep disturbance.
k. Gender reference group: Cisgendered Woman
l. Race reference group: White
m. Ethnicity reference group: Non-Hispanic
n. Specialty reference group: Medical Specialty

© 2025 Tan SF et al. *JAMA Network Open*.

o. Training Level reference group: Intern/PGY1

© 2025 Tan SF et al. *JAMA Network Open*.

eTable 9: Variance Inflation Factor (VIF) Values for Predictor Variables

Variable	Average Work Hours	Work Hours Last Week
Average Work Hours	1.35	—
Work Hours Last Week	—	1.23
Resilience	1.65	1.65
Flourishing	3.27	3.33
Acting Awareness	1.54	1.54
Non-Judgement	1.67	1.66
Loneliness	2.13	2.17
Meaning and Purpose	2.20	2.23
Sleep Disturbance	1.20	1.21
Gender: Cisgender Man	1.09	1.10
Gender: Transgender/Nonbinary	1.04	1.04
Race: Asian	1.09	1.09
Race: Black	1.06	1.07
Race: Multiracial/Other	1.12	1.12
Ethnicity: Hispanic	1.11	1.10
Specialty: Surgery	1.37	1.21
Training level: Mid-level	1.41	1.42
Training level: Senior	1.39	1.40

© 2025 Tan SF et al. *JAMA Network Open*.

eTable 10: Sensitivity Analysis of Association of Work Hours and Burnout, Personal Accomplishment, Stress, and Milestones[a]

Measure	Coefficient, b (95% CI)	Standardized coefficient, β (95% CI)	P-value
**Burnout**			
Average Work Hours	-0.23 (-0.98, 0.52)	-0.03 (-0.13, 0.07)	0.555
Work Hours Last Week	0.08 (-0.39, 0.54)	0.02 (-0.08, 0.11)	0.748
**Personal Accomplishment**			
Average Work Hours	0.17 (-0.10, 0.44)	0.07 (-0.04, 0.17)	0.223
Work Hours Last Week	0.10 (-0.07, 0.27)	0.06 (-0.04, 0.15)	0.236
**Stress**			
Average Work Hours	0.79 (0.12, 1.45)	0.12 (0.02, 0.22)	0.021
Work Hours Last Week	1.14 (0.74, 1.54)	0.26 (0.17, 0.35)	<.001
**Milestones**			
Average Work Hours	0.11 (0.04, 0.18)	0.12 (0.05, 0.19)	0.002
Work Hours Last Week	0.04 (0.00, 0.09)	0.07 (0.00, 0.14)	0.047

a. Sensitivity analysis of linear regression models examining the association between work hours and each outcome variable, excluding participants 3 standard deviations from the mean (n=16). Separate models were run for average hours worked and hours worked in the last week. Coefficients (b) represent unstandardized estimates for the effect per 10-hour increase in weekly work hours (e.g., for every 10-hour increase in average work hours, the Milestones score increased by 0.11). Standardized coefficients (β) allow for comparison of effect sizes across variables.
b. Abbreviated Maslach Burnout Inventory-Human Services Survey (MBI-HSS) Emotional Exhaustion and Depersonalization subscale range: 0-36. Higher scores indicate higher burnout.
c. MBI-HSS Personal Accomplishment subscale range: 0-18. Higher scores indicate higher personal accomplishment and lower burnout.
d. Perceived Stress Scale (PSS) range: 0-40, higher scores indicate greater perceived stress.
e. The Accreditation Council for Graduate Medical Education Milestones range: 1-5, higher scores indicate higher self-assessment of core competencies in Patient Care, Practice-Based Learning and Improvement, Professionalism, and Interpersonal and Communication Skills.

© 2025 Tan SF et al. *JAMA Network Open*.

eFigure 1: Resilience[a] as a Moderator of Average Work Hours and Stress[b] Relationship[c]

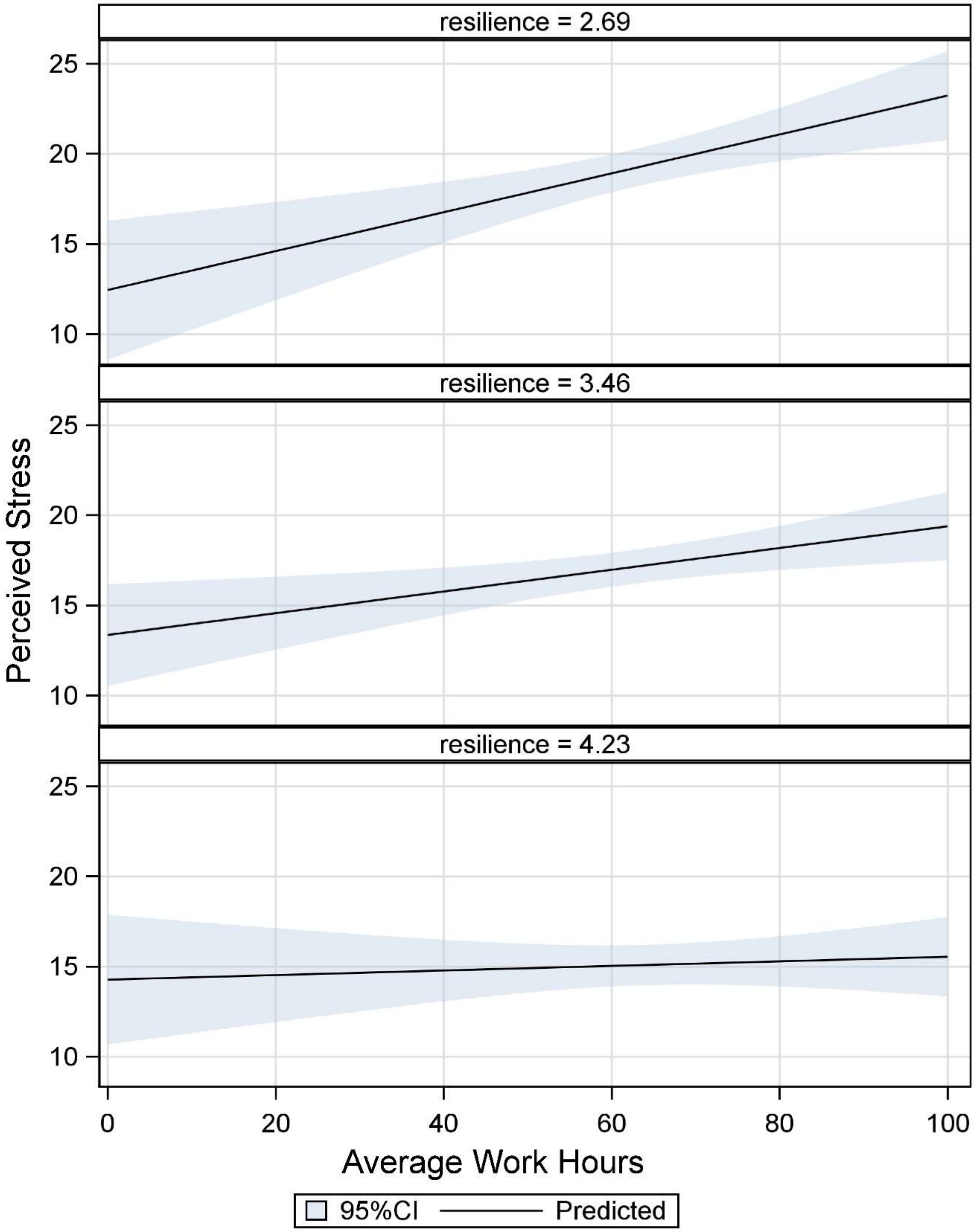

- **a.** Brief Resilience Scale range: 0-6, higher scores indicating higher resilience.
- **b.** Perceived Stress Scale (PSS) range: 0-40, higher scores indicate greater perceived stress.
- **c.** Relationship between work hours and stress across resilience scores (mean - 1 SD, mean, and mean + 1 SD) in multiple linear regression models of average work hours predicting stress while controlling for demographics (gender, ethnicity, specialty, training level, relationship statues, geography, and season of survey completion) and other well-being moderators post Variance Inflation Factor (VIF) Adjustment.

© 2025 Tan SF et al. *JAMA Network Open*.

eFigure 2: Sleep Disturbance[a] as a Moderator of Work Hours and Milestones[b] Relationship[c]

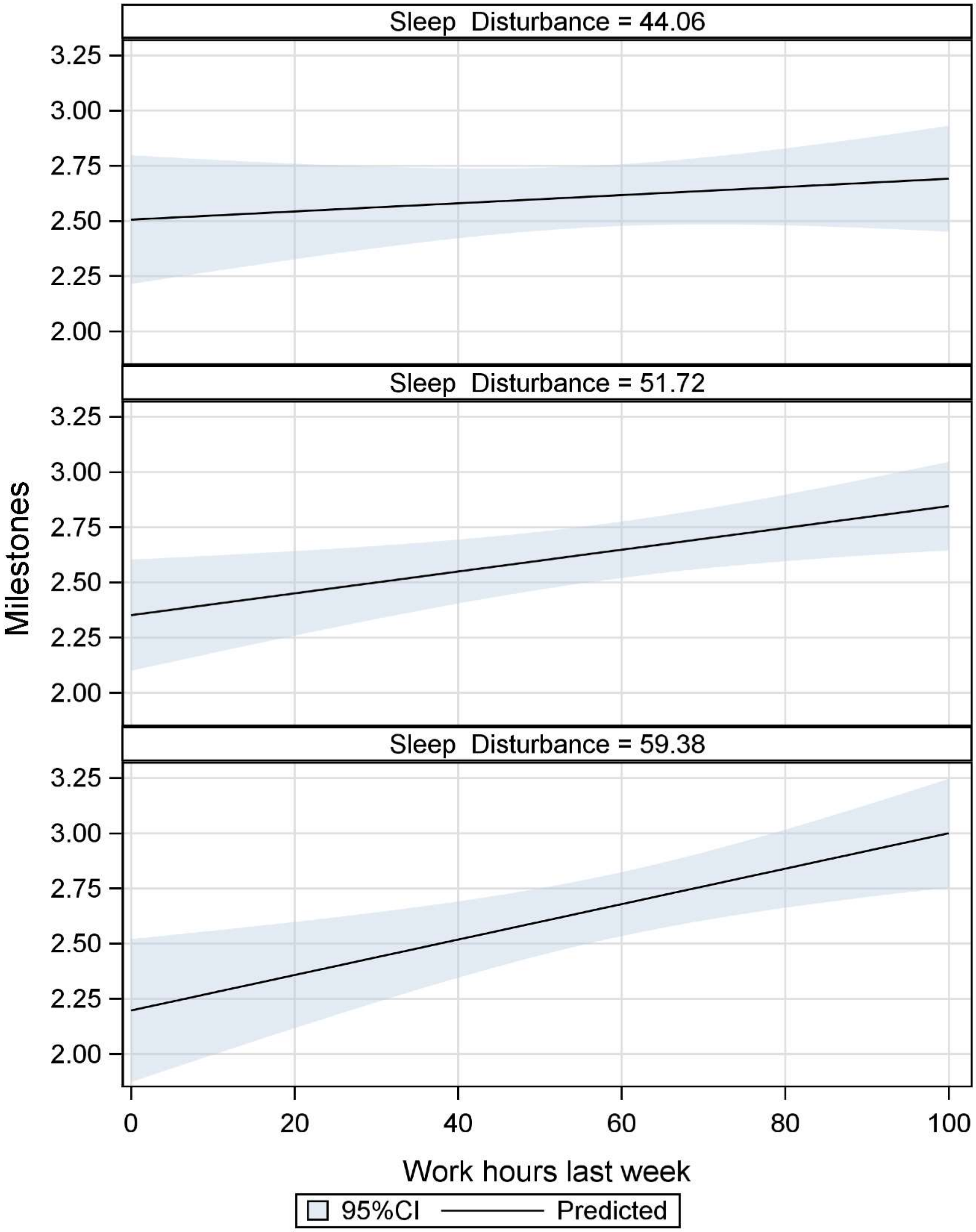

a. PROMIS Sleep Disturbance Scale scored as T-scores (mean = 50, SD = 10) where higher scores indicate more sleep disturbance.
b. The Accreditation Council for Graduate Medical Education Milestones range: 1-5, higher scores indicate higher self-assessment of core competencies in Patient Care, Practice-Based Learning and Improvement, Professionalism, and Interpersonal and Communication Skills.
c. Relationship between work hours last week and predicted self-assessed ACGME competency milestones across sleep disturbance scores ((mean - 1 SD, mean, and mean + 1 SD) in multiple linear regression models of work hours last week predicting self-assessed ACGME competency milestones while controlling for demographics

© 2025 Tan SF et al. *JAMA Network Open*.

(gender, ethnicity, specialty, training level, relationship statues, geography, and season of survey completion) and other well-being moderators post Variance Inflation Factor (VIF) Adjustment.

© 2025 Tan SF et al. *JAMA Network Open*.

eFigure 3: Race as a Moderator of Work Hours Last Week and Milestones[a] Relationship[b]

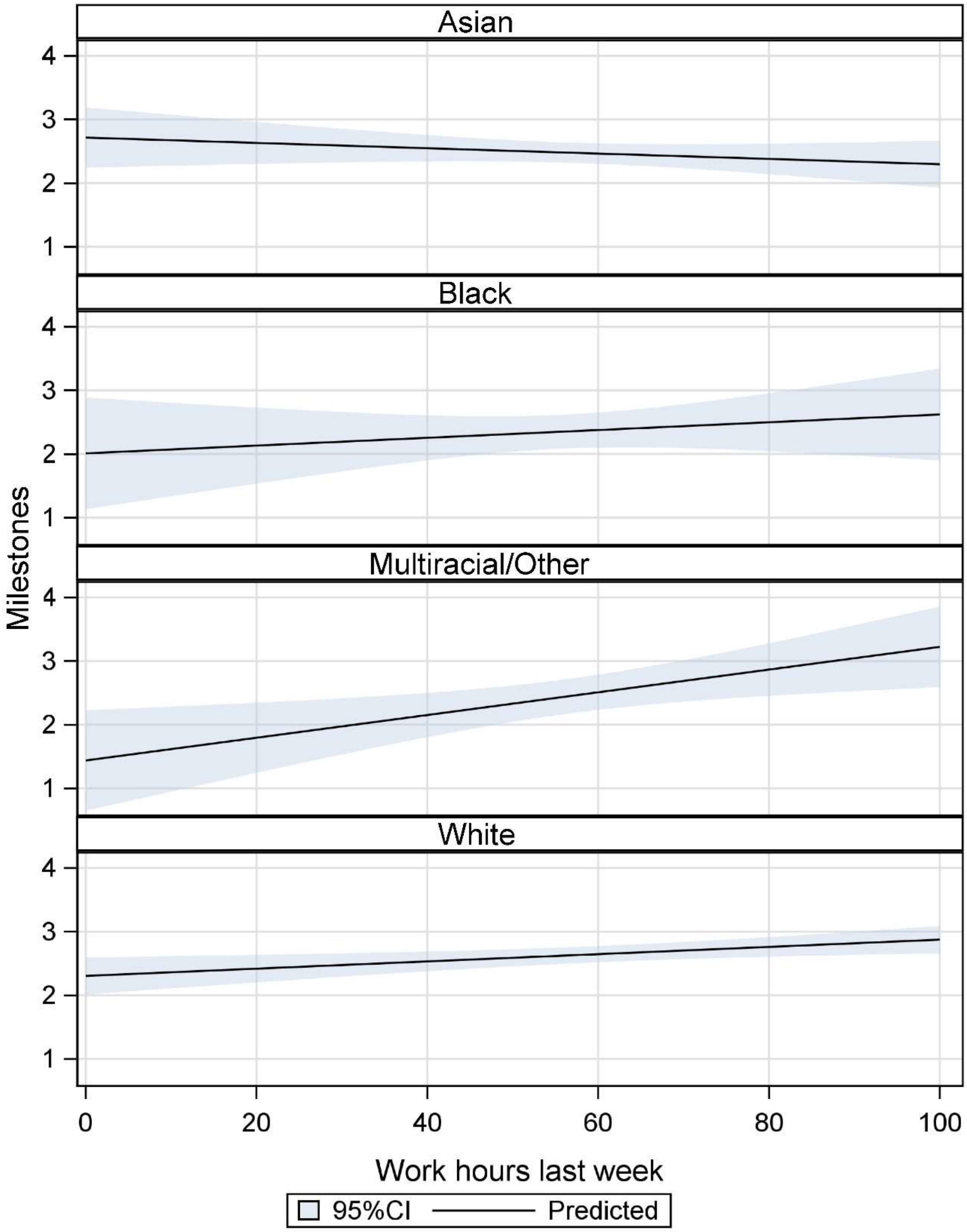

a. The Accreditation Council for Graduate Medical Education Milestones range: 1-5, higher scores indicate higher self-assessment of core competencies in Patient Care, Practice-Based Learning and Improvement, Professionalism, and Interpersonal and Communication Skills.
b. Relationship between work hours last week and predicted self-assessed ACGME competency milestones across racial groups in multiple linear regression models of work hours last week predicting self-assessed ACGME competency milestones while controlling for demographics (gender, ethnicity, specialty, training level, relationship statues, geography, and season of survey completion) and other well-being moderators post Variance Inflation Factor (VIF) Adjustment.

© 2025 Tan SF et al. *JAMA Network Open*.

eAppendix. Survey Instruments

Demographic and work hours survey instrument below. Burnout, well-being and competency outcomes surveyed separately through validated self-report instruments. See methods and reference list for full list of measures.

Demographic and Work Hours Survey

1. What is your specialty?
    - Emergency Medicine
    - Family Medicine
    - Internal Medicine
    - Obstetrics and Gynecology
    - Surgery or surgical subspecialty

2. If surgery, what specialty in surgery?
    - General Surgery
    - Cardiothoracic Surgery
    - Ophthalmology
    - Orthopedic Surgery
    - Otolaryngology
    - Plastics Surgery
    - Urology
    - Vascular Surgery

3. Please select the state in which you are training.

4. What is the name of the residency program you are in? (e.g. University of Wisconsin Hospital and Clinics)

5. What postgraduate year (PGY) are you?

6. What is your age?____

7. Are you Hispanic or Latino? (Hispanic or Latino: A person of Mexican, Puerto Rican, Cuban, South or Central American, or other Spanish culture or origin, regardless of race.)
    - 0 = Not Hispanic or Latino
    - 1 = Yes, Hispanic or Latino

8. Which of the following best describes your race (circle one or more)?
    - 1 = American Indian or Alaska Native
    - 2 = Asian
    - 3 = Black or African American
    - 4 = Native Hawaiian or Other Pacific Islander
    - 5 = White
    - 6 = Other: ______________________________
    - 7 = Prefer not to answer

© 2025 Tan SF et al. *JAMA Network Open*.

9. What sex were you assigned at birth, on your birth certificate?
    - Female
    - Male
    - Not listed, please specify (text box)
    - Prefer not to answer

10. What is your gender? (check all that apply)
    - Man
    - Non-Binary
    - Woman
    - Not listed, please specify (text box)
    - Prefer not to answer

11. Are you transgender?
    - Yes
    - No
    - It's complicated/other
    - Prefer not to answer

12. What is your relationship status?
    - 1 = married
    - 2 = in a long-term, committed relationship
    - 3 = single and never married
    - 4 = separated
    - 5 = divorced
    - 6 = widowed
    - 7 = Other (SPECIFY:____)

13. How many hours do you work during an average week?

14. How many hours did you work last week?

© 2025 Tan SF et al. *JAMA Network Open*.
