# Supplementary material for: Work Hours, Stress, and Burnout Among Resident Physicians
Source: JAMA Netw Open. 2026 Jan 14;9(1):e2553974. doi: 10.1001/jamanetworkopen.2025.53974 (PMC12805447; doi:10.1001/jamanetworkopen.2025.53974)
Supplement: Supplement 2. — Data Sharing Statement [file jamanetwopen-e2553974-s002.pdf]

Data Sharing Statement

Tan. Work Hours, Stress, and Burnout Among Resident Physicians. *JAMA Netw Open*. Published January 14, 2026. doi:10.1001/jamanetworkopen.2025.53974

Data
Data available: Yes
Data types: Deidentified participant data, Data dictionary
How to access data: open science framework files: https://osf.io/zkm4d/files/osfstorage
When available: With publication

Supporting Documents
Document types: Statistical/analytic code
How to access documents: statistical analytical code will be available upon reasonable request from the corresponding author, stan75@wisc.edu.
When available: With publication

Additional Information
Who can access the data: The data is available to the public.
Types of analyses: The data is available to the public.
Mechanisms of data availability: The data is available to the public.
